# Safety profiles of offspring born from early-follicular long-acting GnRH agonist protocol and daily mid-luteal GnRH agonist protocol: a retrospective study

**DOI:** 10.1186/s12884-024-06589-7

**Published:** 2024-05-28

**Authors:** Linqing Du, Jianyuan Song, Wenqian Fan, Tian Ye, Huijuan Kong

**Affiliations:** 1 Center for Reproductive Medicine, The First Affiliated Hospital of Zhengzhou University, No.1, Jianshe Road, Zhengzhou, 450000 China; 2 Henan Key Laboratory of Reproduction and Genetics, The First Affiliated Hospital of Zhengzhou University, Zhengzhou, China; 3 Henan Provincial Obstetrical and Gynecological Diseases (Reproductive Medicine) Clinical Research Center, The First Affiliated Hospital of Zhengzhou University, Zhengzhou, China; 4 Henan Engineering Laboratory of Preimplantation Genetic Diagnosis and Screening, The First Affiliated Hospital of Zhengzhou University, Zhengzhou, China

**Keywords:** Assisted reproductive technology, Early-follicular long-acting GnRH-a long protocol, Luteal phase short-acting GnRH-a long protocol, Congenital anomaly, Neonatal outcomes

## Abstract

**Background:**

The gonadotropin hormone-releasing hormone agonists (GnRH-a) have been widely used for controlled ovarian stimulation in assisted reproductive technology (ART). The early-follicular long-acting GnRH-a long protocol (EFL) and the luteal phase short-acting GnRH-a long protocol (LPS) are commonly used GnRH agonist protocols. We conducted a retrospective analysis to assess and compare the rates of congenital abnormalities and safety profiles in offspring born from the EFL and LPS protocols.

**Methods:**

We conducted a retrospective cohort study to analyze and compare neonatal data from patients who using EFL or LPS protocols at our center between January 1, 2014, and June 30, 2017. The study ultimately included 1810 neonates from 1401 cycles using the EFL protocol and 2700 neonates from 2129 cycles using the LPS protocol.The main outcome measures are gestational age at delivery, birth weight, and congenital anomaly rate.To assess the influence of various factors on congenital abnormalities, a random-effects logistic regression model was employed.

**Results:**

The EFL and LPS protocols led to similar congenital anomaly rates (1.64% vs. 2.35%, *P* = 0.149). No significant differences were found between the two groups regarding birth weight and its categories, newborn gender and congenital anomaly rate. The results of the multivariate logistic regression model indicated no association between congenital anomaly and BMI, duration of infertility, treatment protocol, fertilization method, or embryo transfer stage. Compared with singleton pregnancies, the probability of congenital defects in multiple pregnancies was 2.64 times higher (OR: 2.64, 95% CI: 1.72–4.05, *P* < 0.0001). Newborns with congenital defects were born with a lower gestational age compared with full-term pregnancies.

**Conclusion:**

In conclusion, the EFL protocol is considered a safe option for ensuring offspring safety, comparable with the LPS protocol; however, multiple pregnancies represent an independent risk factor for congenital abnormalities. This approach can be widely adopted; however, prioritizing single embryo transfers is strongly recommended to minimize the potential risks associated with multiple pregnancies in offspring.

## Background

With the continuous development of assisted reproductive technology (ART) and its growing acceptance in society, ART has become a standard and prevalent approach for treating infertility. An increasing number of couples with infertility are conceiving through techniques such as in vitro fertilization (IVF) and intracytoplasmic sperm injection (ICSI). In the year 2019, a total of 1,077,813 treatment cycles were registered in Europe, resulting in 203,665 live births, representing a 7% rise compared to the previous year [1]. There are over 8 million offspring born through ART technology worldwide. Historically, the primary focus of reproductive experts was on improving clinical pregnancy rates and cumulative live birth rates. However, substantial concern and debate remain regarding whether certain artificial extracorporeal procedures and non-physiological interventions, particularly the use of high-dose hormonal medications, may increase the occurrence of adverse obstetric and perinatal outcomes [2–5].

Over the last four decades, gonadotropin hormone-releasing hormone agonists (GnRH-a) have been employed for controlled ovarian stimulation in IVF. Studies have established that GnRH-a down-regulation enhances the synchrony of follicular development and the number of oocytes retrieved. Furthermore, it prevents the premature onset of endogenous luteinizing hormone (LH) peaks, consequently improving embryo quality and clinical pregnancy rates. Following extensive research and development, various ovarian stimulation protocols have been established based on GnRH-a. Among these, the early-follicular long-acting GnRH-a long protocol (EFL) protocol is prominent as a relatively controlled ovarian stimulation (COS) protocol, primarily because it requires a single injection and it is well-tolerated by patients. This protocol is favored among reproductive medicine specialists for its higher clinical pregnancy rates. It is supported by multiple studies, revealing a significant increase in clinical pregnancy rates compared with the luteal phase short-acting GnRH-a long protocol (LPS) and GnRH antagonist protocol [6, 7]. Both PSL and EFL protocols can effectively suppress the pituitary. EFL protocol, using long-acting GnRH-a, provides stronger and longer suppression [8], potentially affecting ovarian response to exogenous FSH, so the EFL protocol needs higher doses of gonadotropins and a longer duration of treatment than PSL protocol. In addition, it has been suggested that reduced LH levels following intense pituitary suppression may have negative implications for oocyte quality [9]. The potential implications of these differences on embryonic and neonatal health remain uncertain. The LPS protocol remains a classic COS protocol and is commonly selected as a control group [10, 11]. Consequently, in this study, we employed LPS as the reference and conducted a retrospective analysis to assess and compare the rates of congenital abnormalities and safety profiles in offspring born from the EFL and LPS protocols.

## Methods

### Study participants and methodology

We conducted a retrospective cohort study to analyze and compare neonatal data from patients who underwent IVF/ICSI using EFL or LPS protocols at our center between January 1, 2014, and June 30, 2017. Patients with specific issues, such as chromosomal abnormalities, single-gene disorders, thyroid diseases, pelvic tuberculosis, and congenital uterine malformations, were excluded from the analysis. Cycles lacking accessible follow-up data were also excluded. The study ultimately included 1810 neonates from 1401 IVF/ICSI cycles using the EFL protocol and 2700 neonates from 2129 IVF/ICSI cycles using the LPS protocol. None of the couples were included multiple times in all cycles.

This study was approved by the Ethics Review Committee of the First Affiliated Hospital of Zhengzhou University. Written informed consent for participation was not required due to the retrospective nature of this study, in accordance with the national legislation and institutional requirements.

### Treatment protocols

In the LPS protocol group, patients underwent transvaginal ultrasound and serum progesterone tests on the 19th − 21st day of their menstrual cycles. If the serum progesterone level > 3 ng/ml, GnRH-a was administered for 14 days for down-regulation (0.1 mg/day for the first 10 days, followed by 0.05 mg/day for the next 4 days). Subsequent evaluation involved transvaginal ultrasound and serum hormone assessments, with down-regulation criteria including follicle-stimulating hormone (FSH) < 5 mIU/mL, LH < 5 mIU/mL, estradiol (E_2_) levels < 30 pg/mL, serum progesterone < 0.6 ng/ml, antral follicle size within 4–7 mm, and endometrial thickness < 5 mm. Once these criteria were met, gonadotropin stimulation commenced, with the Gn dose adjusted based on patient age, baseline anti-Müllerian hormone (AMH) level, and body mass index (BMI). Regular transvaginal ultrasound and serum hormone assessments (LH, E_2_, progesterone) were performed to adjust the Gn dose. When the dominant follicle reached a size of ≥ 20 mm, with another follicle measuring ≥ 18 mm or more than 2/3 of the follicles had a size of ≥ 16 mm, a combination of 250 µg of recombinant human choriogonadotropin alfa and 2000 IU of human chorionic gonadotropin (hCG) was administered on the same evening. Oocyte retrieval was then scheduled 37 h after hCG trigger. Following oocyte retrieval, oral dydrogesterone (20 mg BID) was initiated on the same day, and fertilization of oocyte and sperm occurred in vitro. Embryo transfer into the uterine cavity was performed at the cleavage or blastocyst stage. From the day of transfer, dydrogesterone was reduced to 10 mg TID, with progesterone sustained-release vaginal gel (900 mg qd) or progesterone soft capsules (200 mg BID). Progesterone sustained-release vaginal gel or progesterone soft capsules was discontinued 45 days post-transfer, while dydrogesterone was tapered off gradually over 65 days post-transfer. Regular follow-up visits were made after embryo transfer, with phone-based follow-up for neonatal outcomes after delivery.

In the EFL protocol group, patients underwent transvaginal ultrasound and serum FSH, LH, E2, and progesterone tests on the 2nd and 3rd days of their menstrual cycles. If no substantial follicular growth, cysts, or abnormalities in serum hormone levels were observed, GnRH-a was administered at a dose of 3.75 mg for down-regulation. After 28 days, patients returned to the hospital for repeat transvaginal ultrasound and serum hormone evaluation. Gn dose was adjusted as needed, based on patient age, baseline AMH, and BMI, to facilitate follicular growth. The remaining procedure was performed as in the LPS group.

### Neonatal outcomes

A comprehensive evaluation encompassed all clinical delivery cases. Trained and certified nurses proficient in postnatal follow-up conducted telephone interviews to gather information, including birth-related factors such as gestational age, delivery method, obstetric complications, newborn birth weight, length, and the presence of congenital defects.

### Statistical analysis

Empower software (http://www.empowerstats.com) and R version 4.1.0 (http://www.R-project.org) were employed for all analyses. The normality of continuous variables was assessed with the Shapiro-Wilk test. If the data were normally distributed, the student’s t-test was employed for comparing continuous variables; otherwise, the Kruskal-Wallis test was utilized. Where applicable, Fisher’s exact test or the chi-squared test was used to compare proportions.

To assess the influence of various factors on congenital abnormalities, a random-effects logistic regression model was employed. Potential factors in this model included maternal age, BMI, duration of infertility, the fertilization technique (IVF/ICSI), singleton versus multiple pregnancies, the newborn gender embryo transfer type (cleavage-stage embryo/blastocyst), the number of embryos transferred in each cycle, and the COS protocol (LPS/EFL). The impact of these risk factors on congenital abnormalities was expressed using adjusted odds ratios (OR) and 95% confidence intervals (CI). Differences were considered statistically significant when the *p*-value was < 0.05.

## Results

In our database, 4,123 cycles were identified, comprising 2,512 cycles following the LPS protocol and 1,611 cycles following the EFL protocol. After applying the exclusion criteria, 3,530 cycles resulting in live births were included, including 1810 neonates from 1401 IVF/ICSI cycles using the EFL protocol and 2700 neonates from 2129 IVF/ICSI cycles using the LPS protocol.

Table 1 presents the baseline characteristics of both groups. There were no significant differences in most baseline characteristics between the two groups. However, there were some differences in baseline factors between the two groups, such as antral follicle count, the number of good-quality embryos, gestational age at delivery, and the method of fertilization.

**Table 1:** Basic characteristics of the EFL and LPS groups

Characteristics	EFL protocol(*n* = 1401)	LPS protocol(*n* = 2129)	Standardize diff.	*P*-value
Maternal age (y)	30.17 ± 4.32	30.06 ± 4.51	0.02 (-0.04, 0.09)	0.47
Paternal age (y)	31.27 ± 5.38	31.21 ± 5.38	0.01 (-0.06, 0.08)	0.775
Maternal BMI (kg/m^2^)	22.48 ± 3.11	22.67 ± 3.15	0.06 (-0.00, 0.13)	0.068
Antral follicle count	13.69 ± 6.35	13.14 ± 5.83	0.09 (0.02, 0.16)	0.043
No. of oocytes retrieved	11.78 ± 5.05	11.32 ± 5.23	0.09 (0.02, 0.16)	0.01
Primary infertility	712 (50.97%)	1081 (51.01%)	0.00 (-0.07, 0.07)	0.978
Tubal factor	745 (53.18%)	1069 (50.21%)	0.06 (-0.01, 0.13)	0.085
PCOS	163 (11.63%)	160 (7.52%)	0.14 (0.07, 0.21)	< 0.001
Male primary infertility	769 (55.44%)	1161 (54.89%)	0.01 (-0.06, 0.08)	0.749
Fertilization method			0.12 (0.05, 0.19)	< 0.001
IVF	1021 (72.88%)	1436 (67.45%)		
ICSI	380 (27.12%)	693 (32.55%)		
No. of transferred embryos	1.90 ± 0.30	1.91 ± 0.42	0.01 (-0.06, 0.08)	0.793
Embryo transfer stage			0.09 (0.02, 0.16)	0.011
Cleavage-stage embryo	1279 (91.29%)	1887 (88.63%)		
Blastocyst	122 (8.71%)	242 (11.37%)		
Gestational age at delivery (wk)	38.52 ± 1.63	38.85 ± 1.68	0.19 (0.11, 0.28)	< 0.001
Time of delivery (wk)				
<36	215 (15.74%)	255 (12.18%)	0.10 (0.03, 0.17)	0.003
37–41	1119 (81.92%)	1793 (85.63%)	0.10 (0.03, 0.17)	0.004
≥ 42	0 (0.00%)	14 (0.67%)	0.12 (0.05, 0.18)	0.002
Delivery mode			0.05 (-0.01, 0.12)	0.117
vaginal delivery	293 (21.45%)	405 (19.27%)		
cesarean delivery	1073 (78.55%)	1697 (80.73%)		
Number of deliveries			0.05 (-0.02, 0.12)	0.127
Singletons	995 (71.02%)	1562 (73.37%)		
Multiples	406 (28.98%)	567 (26.63%)		
Congenital malformation	23 (1.64%)	50 (2.35%)	0.05 (-0.02, 0.12)	0.149
GDM	44 (3.14%)	19 (0.89%)	0.16 (0.09, 0.23)	< 0.001

Note: Data are presented as mean ± SD for continuous variables or n (%) for categorical variables

BMI = body mass index; PCOS = polycystic ovary syndrome; ICSI = intracytoplasmic sperm injection; IVF = in vitro fertilization; GDM = gestational diabetes mellitus

In analyzing birth characteristics reflecting neonatal growth and development indicators such as birth weight, gestational age at birth, and the presence of congenital defects, births were categorized as singletons and multiples (twins and higher-order multiples). This distinction was made because multiples are closely associated with ART and congenital defects [12]. Higher-order multiples were infrequent at our center, even in cases resulting from ART, and were therefore not separately analyzed. Table 2 presents the neonatal outcome indicators for both protocols in singleton and multiple birth cycles. In singleton birth cycles (EFL protocol (*n* = 995) vs. LPS protocol (*n* = 1562)), significant differences were observed between the two groups in terms of gestational weeks at delivery (38.52 ± 1.63 vs. 38.85 ± 1.68; *P* < 0.001) and newborn length (50.43 ± 2.05 vs. 49.76 ± 2.65; *P* = 0.013). In multiple birth cycles (EFL protocol (*n* = 815) vs. LPS protocol (*n* = 1138)), significant differences were observed in gestational weeks at delivery (36.12 ± 2.36 vs. 36.69 ± 2.19; *P* < 0.001), number of full-term deliveries (444 (54.48%) vs. 739 (64.94%); *P*<0.001), mall for gestational age (SGA) (175 (22.01%) vs. 298 (26.58%); *P*<0.022) between the two groups. However, no significant differences existed in birth gender, delivery method, congenital anomalies, or birth weight between the two groups.

**Table 2:** Characteristics of the neonate in the EFL protocol group and PSL protocol group

Birth characteristic	Singleton births					Multiple births			
	EFL protocol (*n* = 995)	LPS protocol (*n* = 1562)	Standardize diff.	*P*-value		EFL protocol (*n* = 815)	LPS protocol (*n* = 1138)	Standardize diff.	*P*-value
Gestational age at delivery(wk)	38.52 ± 1.63	38.85 ± 1.68	0.19 (0.11, 0.28)	**< 0.001**		36.12 ± 2.36	36.69 ± 2.19	0.25 (0.16, 0.34)	**< 0.001**
Full-term birth	897 (90.15%)	1425 (91.23%)	0.04 (-0.04, 0.12)	0.375		444 (54.48%)	739 (64.94%)	0.21 (0.12, 0.30)	**< 0.001**
Newborn gender			0.01 (-0.07, 0.09)	0.831				0.01 (-0.08, 0.10)	0.752
Female	483 (48.54%)	765 (48.98%)				393 (48.22%)	557 (48.95%)		
Male	512 (51.46%)	797 (51.02%)				422 (51.78%)	581 (51.05%)		
Sex ratio, male/female	1.06	1.04				1.07	1.04		
Birth weight (g)	3375.56 ± 523.19	3382.30 ± 526.38	0.01 (-0.07, 0.09)	0.755		2519.45 ± 507.77	2560.56 ± 491.20	0.08 (-0.01, 0.17)	0.075
Type of birth weight			0.05 (-0.03, 0.13)	0.662				0.12 (0.03, 0.21)	0.11
normal birth weight	840 (87.50%)	1322 (86.63%)				478 (59.82%)	705 (63.46%)		
LBW (< 2,500 g)	39 (4.06%)	58 (3.80%)				286 (35.79%)	368 (33.12%)		
VLBW (< 1,500 g)	3 (0.31%)	9 (0.59%)				35 (4.38%)	35 (3.15%)		
Macrosomia (> 4,000 g)	78 (8.12%)	137 (8.98%)				0 (0.00%)	3 (0.27%)		
Birth length(cm)	50.43 ± 2.05	49.76 ± 2.65	0.28 (0.04, 0.52)	**0.013**		47.69 ± 3.50	47.47 ± 3.08	0.07 (-0.23, 0.36)	0.674
SGA (< 10th percentile)	29 (3.01%)	67 (4.37%)	0.07 (-0.01, 0.15)	0.083		175 (22.01%)	298 (26.58%)	0.11 (0.02, 0.20)	**0.022**
AGA (10th ~ 90th percentile)	658 (68.19%)	1067 (69.65%)	0.03 (-0.05, 0.11)	0.442		602 (75.72%)	810 (72.26%)	0.08 (-0.01, 0.17)	0.09
LGA (> 90th percentile)	278 (28.81%)	398 (25.98%)	0.06 (-0.02, 0.14)	0.121		18 (2.26%)	13 (1.16%)	0.09 (-0.01, 0.18)	0.059
Congenital malformation	10 (1.01%)	31 (1.98%)	0.08 (0.00, 0.16)	0.055		16 (1.96%)	28 (2.46%)	0.03 (-0.06, 0.12)	0.465

Note: Data are presented as mean ± SD for continuous variables or n (%) for categorical variables

LBW = low birth weight; VLBW = very low birth weight; SGA = small for gestational age; AGA = appropriate for gestational age, LGA = large for gestational age

Per the definitions in the International Classification of Diseases, of the 4,510 live-born infants, 85 (1.88%) exhibited congenital defects. Within the LPS protocol group comprising 2,700 infants, 59 (2.19%) had defects, while in the EFL protocol group with 1,810 infants, 26 (1.44%) had defects (Table 3). The difference between the groups was not significant (*P* = 0.07). Comparisons of congenital defects across singleton and multiple pregnancies and between male and female newborns yielded no significant differences. Detailed information regarding defects detected in different organ systems is outlined in Table 3. In both groups, circulatory system diseases were the most frequent congenital defects (23 cases in the LPS group and 9 cases in the EFL group). The second most common defect was musculoskeletal system diseases (7 cases in the LPS and 4 cases in EFL protocol group), followed by digestive system diseases (5 cases in the LPS and 6 cases in EFL group).

**Table 3:** Incidence of congenital malformation in neonate and classification of malformations according to the international classification of diseases, tenth edition

		EFL protocol(*n* = 1810)	LPS protocol(*n* = 2700)	Standardize diff.	*P*-value
	Newborn gender			0.01 (-0.05, 0.07)	0.71
	Female	876 (48.40%)	1322 (48.96%)		
	Male	934 (51.60%)	1378 (51.04%)		
	Number of deliveries (n)			0.06 (-0.00, 0.12)	0.056
	Singletons	995 (54.97%)	1562 (57.85%)		
	Multiples	815 (45.03%)	1138 (42.15%)		
	Congenital malformation	26 (1.44%)	59 (2.19%)	0.06 (-0.00, 0.12)	0.07
Q00-Q07	nervous system	3(0.16%)	4(0.15%)		
Q10-Q18	eye, ear, face, and neck	0	2(0.07%)		
Q20-Q28	circulatory system	9(0.50%)	23(0.85%)		
Q30-Q34	respiratory system	1(0.06%)	6(0.22%)		
Q35-Q37	cleft lip and cleft palate	0	2(0.07%)		
Q38-Q45	digestive system	6(0.33%)	5(0.19%)		
Q50-Q56	genital organs	1(0.06%)	7(0.26%)		
Q60-Q64	urinary system	1(0.06%)	1(0.04%)		
Q65-Q79	musculoskeletal system	4(0.22%)	7(0.26%)		
	Multiple congenital malformation	1(0.06%)	2(0.07%)		

Note: Data are presented as n (%) for categorical variables

**Fig. 1:**
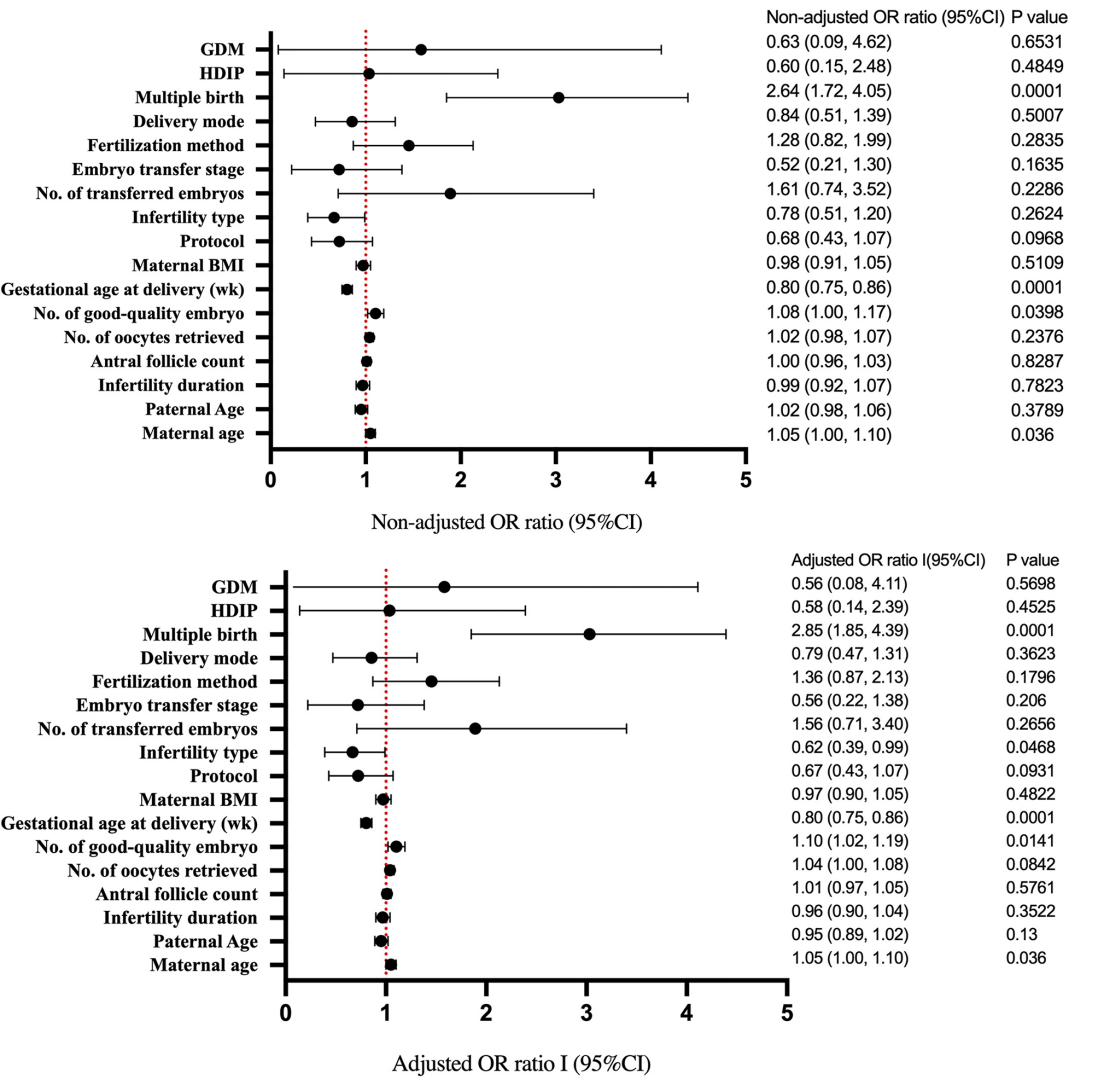
Random effects logistic regression of congenital malformation in neonate Note: Results in table: OR (95%CI) Pvalue. CI = confidence interval; OR = odds ratio; BMI = body mass index; HDIP = Hypertensive Disorders in Pregnancy; GDM = gestational diabetes mellitus Outcomes: Congenital malformation Adjust I adjust for: Maternal age

Table 4; Fig. 1 present the results of a multivariate logistic regression model for factors that may impact congenital defects in newborns. The results indicate no association between congenital defects and BMI, duration of infertility, treatment protocol, fertilization method, or the stage of embryo transfer. However, multiple pregnancies and preterm births significantly increase the likelihood of congenital defects. Compared with singleton pregnancies, the probability of congenital defects in multiple pregnancies was 2.64 times higher (OR: 2.64, 95% CI: 1.72, 4.05, *P* < 0.0001). Newborns with congenital defects were born with a lower gestational age compared with full-term pregnancies.

**Table 4:** Random effects logistic regression of congenital malformation in neonate

	Non-adjusted	Adjust I
Maternal age	1.05 (1.00, 1.10) 0.0360	1.05 (1.00, 1.10) 0.0360
Paternal Age	1.02 (0.98, 1.06) 0.3789	0.95 (0.89, 1.02) 0.1300
Infertility duration	0.99 (0.92, 1.07) 0.7823	0.96 (0.90, 1.04) 0.3522
Antral follicle count	1.00 (0.96, 1.03) 0.8287	1.01 (0.97, 1.05) 0.5761
No. of oocytes retrieved	1.02 (0.98, 1.07) 0.2376	1.04 (1.00, 1.08) 0.0842
No. of good-quality embryo	1.08 (1.00, 1.17) 0.0398	1.10 (1.02, 1.19) 0.0141
Gestational age at delivery (wk)	0.80 (0.75, 0.86) < 0.0001	0.80 (0.75, 0.86) < 0.0001
Maternal BMI	0.98 (0.91, 1.05) 0.5109	0.97 (0.90, 1.05) 0.4822
Protocol	0.68 (0.43, 1.07) 0.0968	0.67 (0.43, 1.07) 0.0931
Infertility type	0.78 (0.51, 1.20) 0.2624	0.62 (0.39, 0.99) 0.0468
No. of transferred embryos	1.61 (0.74, 3.52) 0.2286	1.56 (0.71, 3.40) 0.2656
Embryo transfer stage	0.52 (0.21, 1.30) 0.1635	0.56 (0.22, 1.38) 0.2060
Fertilization method	1.28 (0.82, 1.99) 0.2835	1.36 (0.87, 2.13) 0.1796
Delivery mode	0.84 (0.51, 1.39) 0.5007	0.79 (0.47, 1.31) 0.3623
Multiple birth	2.64 (1.72, 4.05) < 0.0001	2.85 (1.85, 4.39) < 0.0001
HDIP	0.60 (0.15, 2.48) 0.4849	0.58 (0.14, 2.39) 0.4525
GDM	0.63 (0.09, 4.62) 0.6531	0.56 (0.08, 4.11) 0.5698

Note: Results in table: OR (95%CI) Pvalue. CI = confidence interval; OR = odds ratio; BMI = body mass index; HDIP = Hypertensive Disorders in Pregnancy; GDM = gestational diabetes mellitus

Outcomes: Congenital malformation

Adjust I adjust for: Maternal age

## Discussion

In this study, we investigated neonatal outcomes and congenital defects after the EFL protocol for IVF, using the LPS protocol as a reference. Our findings indicated no significant differences in congenital anomalies, birth gender, delivery mode, or birth weight and its categories between the groups. The results of the variable logistic regression model emphasized that multiple pregnancies and preterm births significantly elevated the risk of congenital anomalies. The likelihood of congenital defects in multiple pregnancies was 2.64 times higher compared to singleton pregnancies. Additionally, neonates born from multiple pregnancies exhibited lower birth weights than those born at term. Our results suggest that the neonatal safety profiles of the EFL and LPS protocols are comparable, while multiple pregnancies independently pose a risk for congenital anomalies.

Offspring safety is a pivotal criterion in assessing COS protocols within ART and is garnering increasing attention. Since its introduction, LPS protocol have gained widespread adoption in clinical practice. Numerous clinical studies have investigated their efficacy and safety. Existing research has affirmed that compared to non-downregulated protocols, the newborn health outcomes in the short-acting GnRH-a exposure group are equal to those in the control group [13]. Consequently, GnRH-a protocols can be considered a standard for evaluating the safety of the EFL protocol.

The clinical pregnancy rate of the EFL protocol is not inferior to that of the antagonist protocol or the LPS long protocol, and EFL protocol may even result in higher live birth rates in fresh embryo transfer cycles [7]. Ovarian stimulations were conducted following pituitary downregulation in both the EFL and PSL protocols. In the EFL protocol, the ues of long-acting GnRH-a during the follicular phase effectively suppressed the secretion of endogenous FSH and LH in patients, leading to more exogenous FSH for ovarian stimulation and lower hormone levels compared to those undergoing the PSL protocol. Despite variations in endocrine profiles between the two protocols, research on the safety of offspring of them remains relatively scarce. In a study conducted at our center in 2022, 1,179 patients with polycystic ovary syndrome (PCOS) who underwent the EFL protocol were compared with 2,390 patients with PCOS who underwent the LPS protocol. The EFL protocol group had higher rates of fresh embryo transfer, clinical pregnancy, and live births than the LPS protocol group. The incidence of gestational diabetes mellitus (GDM) in pregnant mothers in both protocol groups differed significantly, with the LPS protocol group exhibiting a lower incidence than the EFL protocol group (1.42% vs. 5.08%). However, no significant difference was observed in the occurrence of congenital anomalies in the offspring between both groups [11]. Our study utilized a more extensive dataset of live birth, including 1,810 newborns from the LPS protocol and 4100 newborns from the EFL protocol, with 160 and 163 patients with PCOS in each group, respectively. Similarly, we observed a higher incidence of GDM (0.89% vs. 3.14%) in the EFL protocol group than in the LPS protocol group, consistent with Professor Zhai’s findings. Professor Zhai’s analysis suggested that the stimulation protocol was an independent risk factor for GDM, and the differences in the incidence of GDM between the two protocols may be attributed to the use of different drugs and dosages during ovulation induction. During this period, the use of long-acting GnRH-a may lead to glucose intolerance and increased insulin resistance, which could result in an increased risk of GDM in the EFL group [13]. Research has indicated a significant difference in GDM incidence based on GnRH dosage, and using GnRH is a risk factor for GDM [14]. In the EFL protocol group, compared with the LPS protocol group, the more profound downregulation and lower initial Gn dosage led to an increased total Gn dosage and a longer duration of administration during ovulation induction, which may explain the increased incidence of GDM. There was a higher proportion of non-PCOS patients in our study, which resulted in a lower proportion of GDM than in Prof. Zhai’s study.

Another study focused on patients with normal ovarian responses, involving 1,193 patients using the LPS protocol and 2,851 patients using the EFL protocol. The study revealed no differences in the incidence of congenital anomalies between the two groups, both before and after matching, possibly due to the characteristic of lower E2 levels associated with the EFL protocol [15]. Consistent with their findings, our study revealed no significant difference in the incidence of congenital anomalies between the two groups, (2.35% in LPS vs. 1.64% in EFL). A large-scale meta-analysis demonstrated that children born with ART have a 30–40% increased risk of birth defects than naturally conceived children [5]. For instance, in Canada, the Netherlands, and Australia, the prevalence of birth defects resulting from ART is 3.4%, 3.2%, and 8.2% (18) [16, 17], respectively. In our study, the overall incidence of congenital anomalies in live-born infants was 2.068%. Similarly, a population-based study carried out in Shanghai, China, revealed that the incidence of birth defects among ART offspring in the Chinese population is 19.73 per 10,000 live births [18]. Although the results of our study on the rate of congenital malformations were similar to those of previous studies in China., we observed a relatively higher incidence of congenital anomalies than in the previous two studies. Professor Zhai’s study had an average patient age of 28.8 years and 28.7 years in two groups, which is younger than our study population. Furthermore, Professor Zhang’s group included a population with superior ovarian reserves than our study, while our study involved patients with lower ovarian reserves. These differences may explain the higher incidence of congenital anomalies observed in our study.

While Professor Zhang’s study included data on birth defects, it was not the primary outcome, and the study did not provide information regarding the types and severity of those birth defects. Through further research, our study offered a exhaustive examination of parameters that gauge offspring safety, encompassing both the incidence and specific categories of congenital anomalies. Our research revealed that among all types of congenital abnormalities, cardiovascular anomalies were the most prevalent birth defects, consistent with Dr. Kuang’s research [19]. As a recent study suggests, cardiovascular changes are the primary type of functional disorders in IVF offspring [20]. We observed defects such as atrial septal defects, ventricular septal defects, and arteriovenous malformations, all of which are cardiovascular abnormalities. The cause of this congenital anomaly is not entirely clear. Previous research has indicated that exogenous gonadotropins used in ovarian stimulation may exhibit adverse effects on oocyte development, embryo quality, endometrial receptivity, and perinatal outcomes. In mouse models, ovarian hyperstimulation can affect the genomic imprinting of both maternal and paternal genes [21]. A study in 2018 suggested signs of cardiac remodeling and functional impairment in ART twins [22]. The higher rate of twins in ART pregnancies compared with natural pregnancies may contribute to the higher incidence of circulatory system anomalies. Additionally, digestive system anomalies, musculoskeletal system congenital defects and deformities, and genital organ congenital defects were also relatively common. The rate of reproductive system anomalies appeared lower in the EFL group, although this could have been influenced by the limited sample size. Therefore, we cannot conclude that the EFL protocol protects against reproductive system anomalies based on the data.

Regarding newborn weight-related indicators such as low birth weight, macrosomia, appropriate for gestational age (AGA), small for gestational age (SGA) and large for gestational age (LGA), our research findings indicated no significant differences between the two groups. However, when comparing the EFL protocol to the LPS protocol, we observed a lower rate of full-term deliveries. Preliminary clinical parameter analysis revealed that the EFL protocol group exhibited a slightly lower average number of embryos transferred (1.91 ± 0.42 vs. 1.90 ± 0.30 ,*P* = 0.793), and a minor increase in multiple pregnancies (26.63% vs. 28.98%,*P* = 0.127), although these differences were not significant. Subsequently, during the subgroup analysis for single and multiple births, the differences in full-term deliveries between the two groups disappeared. While a significant difference was observed, the actual difference in gestational weeks was minimal (38.85 weeks in the EFL protocol group vs. 38.52 weeks in the LPS protocol group). This suggests that the observed difference lacks clinical significance. In the multiple birth group, the rate of preterm births remained slightly higher in the EFL protocol. This could be attributed to the higher average number of embryos transferred and increased endometrial receptivity during the follicular phase, leading to a higher incidence of high-order multiple pregnancies. Our logistic regression analysis indicated that multiple pregnancies were an independent risk factor for congenital abnormalities, consistent with previous research (26). Multiple pregnancies pose a higher risk of maternal morbidity and are associated with a greater likelihood of perinatal morbidity and mortality. Furthermore, multiple pregnancies are more susceptible to congenital abnormalities than singleton pregnancies [23]. The significantly increased risk of congenital abnormalities in multiple pregnancies can be explained from several perspectives. First, adequate nutrition is a pivotal factor for the normal development of fetuses. Maternal malnutrition may lead to delayed fetal growth or render fetuses more susceptible to factors related to congenital abnormalities and less resilient. Second, shared genetic backgrounds and a crowded uterine environment have been associated with a higher proportion of mechanical deformities in fetuses in multiple pregnancies. In summary, the increased occurrence of congenital abnormalities in multiple pregnancies is likely due to the intricate interplay of various factors, which play a significant role in the development of these congenital abnormalities.

Our study had some limitations. This study specifically emphasized assessing the safety of the EFL protocol for offspring. Earlier research with a similar focus has explored outcomes in individuals with PCOS and those who exhibit a normal response to the protocol. Research on offspring safety suggests that a low response to fertilization is an independent risk factor for an increased incidence of congenital abnormalities [24]. The impact of utilizing different protocols within low-response populations remains an area yet to be explored. Therefore, future investigations may delve deeper into the benefits for diverse population groups.

In conclusion, the EFL protocol is considered a safe option for ensuring offspring safety, comparable with the LPS protocol; however, multiple pregnancies represent an independent risk factor for congenital abnormalities. This approach can be widely adopted; however, prioritizing single embryo transfers is strongly recommended to minimize the potential risks associated with multiple pregnancies in offspring.

## Data Availability

The datasets used and analysed during the current study available from the corresponding author on reasonable request.

## Abbreviations

IVF: in vitro fertilization
ICSI: intracytoplasmic sperm injection
ART: assisted reproductive technology
EFL: early-follicular long-acting GnRH-a long protocol
LPS: luteal phase short-acting GnRH-a long protocol
GnRH-a: gonadotropin hormone-releasing hormone agonist
LH: luteinizing hormone
COS: controlled ovarian stimulation
FSH: follicle- stimulating hormone
E_2_: estradiol
AMH: anti-Müllerian hormone
BMI: body mass index
hCG: human chorionic gonadotropin
OR: odds ratio
CI: confidence interval
PCOS: polycystic ovary syndrome
GDM: gestational diabetes mellitus
AGA: appropriate for gestational age
SGA: small for gestational age
LGA: large for gestational age
LBW: low birth weight
VLBW: very low birth weight
HDIP: Hypertensive Disorders in Pregnancy
